# Supplementary material for: Single Cell RNA-Sequence Analyses Reveal Uniquely Expressed Genes and Heterogeneous Immune Cell Involvement in the Rat Model of Intervertebral Disc Degeneration
Source: Appl Sci (Basel). Author manuscript; Available in PMC 2022 Nov 29. (PMC9706593; doi:10.3390/app12168244)
Supplement: Supplemental file [file NIHMS1845035-supplement-Supplemental_file.pdf]

Single Cell RNA-Sequence Analyses Reveal Uniquely Expressed Genes and Heterogeneous Immune Cell Involvement in the Rat Model of Intervertebral Disc Degeneration
Supplemental Data—August 11, 2022

Milad Rohanifar [1], Sade W. Clayton [2], Garrett W.D. Easson [2], Deepanjali S. Patil [1], Frank Lee [1], Liufang Jing [1], Marcos N. Barcellona [1], Julie E. Speer [1], Jordan J. Stivers [2], Simon Y. Tang [2] and Lori A. Setton [1,2,*]

[1] Department of Biomedical Engineering, Washington University in St. Louis, St. Louis, MO 63130, USA

[2] Department of Orthopedic Surgery, Washington University School of Medicine, St. Louis, MO 63110, USA

Corresponding: setton@wustl.edu

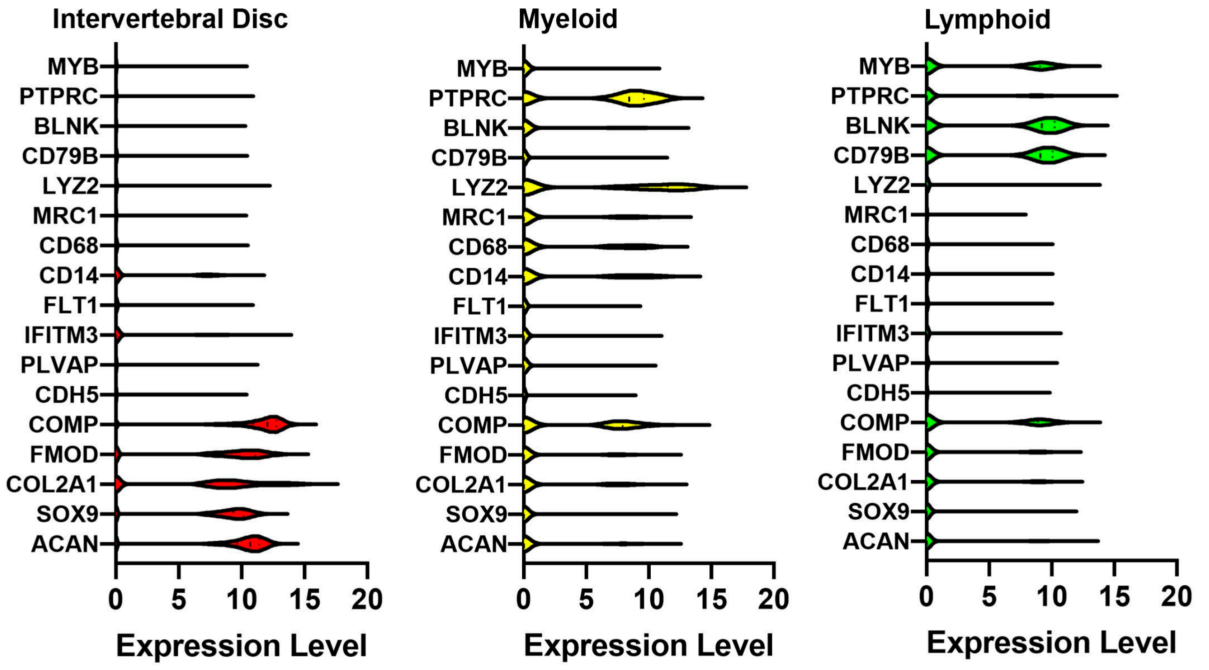

Figure S1. Expression level of marker genes within intervertebral disc cells, myeloid, and lymphoid cells from CON samples at 8 weeks post-surgery.

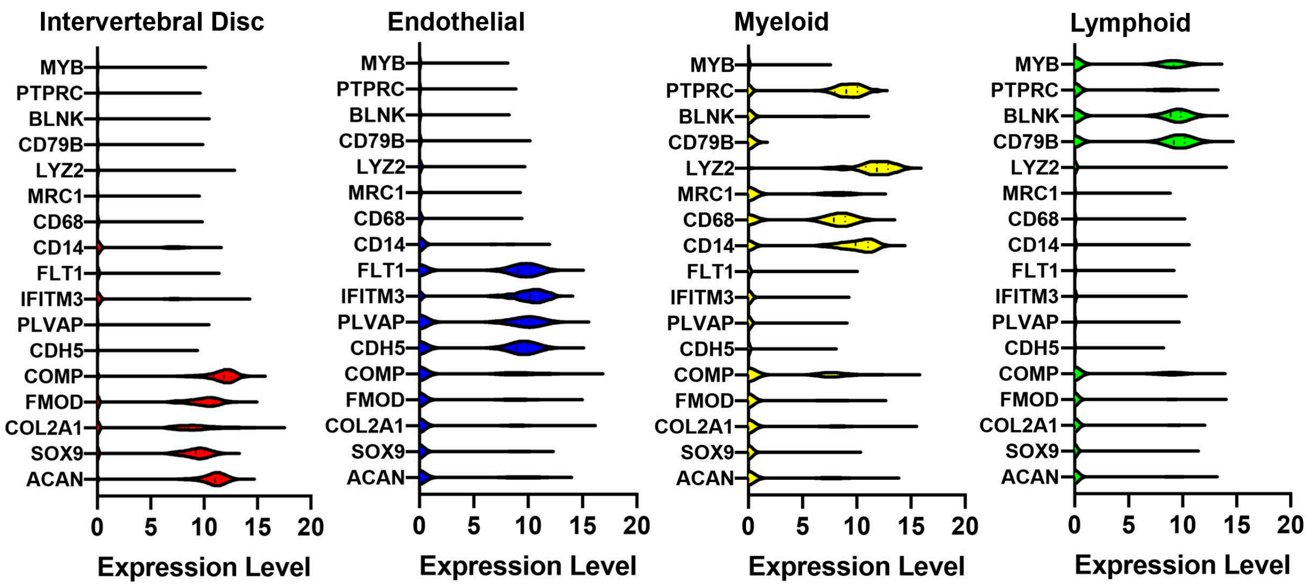

Figure S2. Expression level of marker genes within intervertebral disc cells, endothelial cells, myeloid, and lymphoid cells from LDP samples at 8 weeks post-surgery.
